# Supplementary material for: HexFire: A Flexible and Accessible Wildfire Simulator
Source: Land (Basel). Author manuscript; Available in PMC 2023 Aug 11. (PMC9534040; doi:10.3390/land11081288)
Supplement: Supplement1 [file NIHMS1830362-supplement-Supplement1.docx]

**Supplemental Information**

**HexFire: A Flexible and Accessible Wildfire Simulator**

**Nathan H. Schumaker 1,* , Sydney M.Watkins 2 and Julie A. Heinrichs 3**

- **Externally hosted supplementary file 1**
  Doi: https://doi.org/10.3390/land11081288
  Link: [**https://youtu.be/ p_ILu2zXDAI**](https://youtu.be/%20p_ILu2zXDAI)
  Description: *A 40-min video tutorial about HexFire*
- **Externally hosted supplementary file 2**
  Doi: https://doi.org/10.3390/land11081288
  Link: [**http://www.hexsim.net**](http://www.hexsim.net/)
  Description: *HexSim, HexFire, and all of the content associated with the three examples*
